# A case of LGL leukemia with paucity of erythropoiesis

**DOI:** 10.1002/ccr3.3170

**Published:** 2020-07-29

**Authors:** Habib M. Razavi

**Affiliations:** ^1^ Division of Hematopathology and Transfusion Medicine Fraser Health Authority and Department of Pathology and Laboratory Medicine University of British Columbia Vancouver BC Canada

**Keywords:** LGL leukemia, neutropenia, pure red cell aplasia

## Abstract

The differential diagnosis for neutropenia is large and includes, drug effect, viral infections, sepsis, immune, hypersplenism, and bone marrow disorder. The presence of an autoimmune disorder and large granular lymphocytosis should prompt assessment for immunophenotyping and in select cases a bone marrow biopsy to rule out LGL‐leukemia.

## Graphical abstract

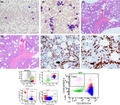

A 63‐year‐old male patient with progressive lymphocytosis and cytopenias is described. At presentation, he complained of an upper respiratory tract infection and infrequent oral ulcers. His CBC showed a hemoglobin at 106 g/L, neutrophils at 0.9 × 10E9/L, and lymphocytes at 16.2 × 10E9/L. The peripheral blood film showed lymphocytosis with LGL morphology (Figure,1 Panel A, ×20). Flow cytometric immunophenotyping of the peripheral blood showed an abnormal CD8 + population with the LGL immunophenotype (not shown). A bone marrow aspirate showed trilinear hematopoiesis with paucity of erythropoiesis. As well, a lymphoid infiltrate was present showing LGLs (Panel B × 50). Biopsy showed a hypercellular bone marrow with left shifted myelopoiesis, megakaryocytopoiesis, and scant erythropoiesis (H&E, panels C × 10, and panel D × 40, respectively). CD 3 showed interstitial and intersinusoidal (black arrow) infiltration of T cells (panel E, ×10 and panel F, ×40). Flow cytometric immunophenotyping of the bone marrow aspirate showed an expansion of CD8 + population co‐expressing CD57 (lower panels, flow cytometry plots). The sequential gating shows the lymphocyte gate in the side scatter versus CD45 plot followed by CD2 +, CD3+ lymphocytes which show expansion of the CD8 compartment (91.7% of the lymph gate) co‐expressing CD57 (LGL phenotype). In contrast, CD3‐CD56 + NK cells accounted for 1% of the lymphocyte gate and 0.2% of total events (purple events, bottom right panel). This distinction is important as NK LGL disorders can clinically mimic T‐LGL disease. Moreover, this is not a polyclonal expansion of reactive T LGLs. Multiplex PCR analysis showed a clonal rearrangement in the TCR beta gene. The diagnosis of LGL leukemia with pure red cell aplasia was made. The differential diagnosis of neutropenia is large. In cases where reactive causes of neutropenia are excluded, and lymphocytosis is present, a logical flow to investigation may include blood film morphology, peripheral blood immunophenotyping, TCR/BCR/KIR rearrangement studies, and in selected cases a bone marrow biopsy.^1^ The close communication of the hematopathologist and the hematologist is key in properly diagnosing this relatively rare disorder.

**Figure 1:**
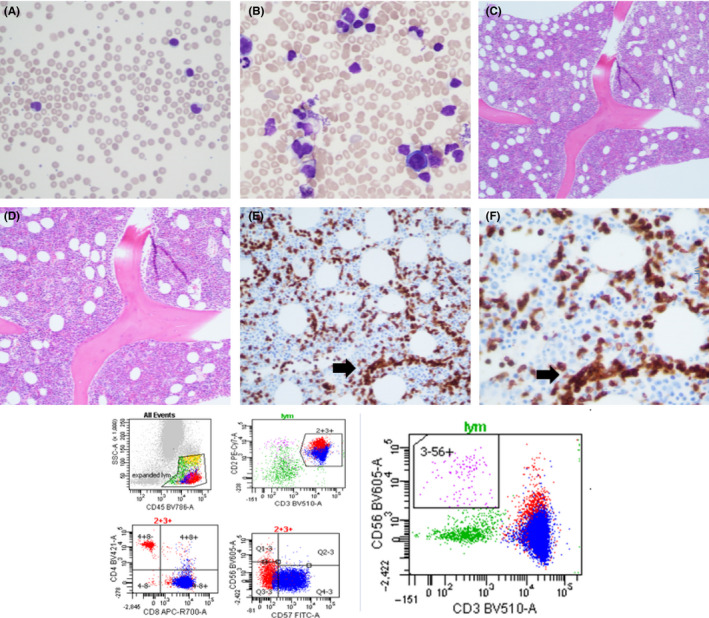
Interstitial and intrasinusoidal leukemic infiltration of CD3 + cells that by flow cytometry showed co‐expression of CD8 and CD57. These cells were clonal by TCR rearrangement studies in keeping with LGL leukemia. Please see text for details

## Conflict of interest

None declared.

## Author Contribution

HMR is the case pathologist, took all the photographs, prepared the manuscript, and diagnosed the case. There are no other authors on this submission.
